# Colonic Perforation in Polyarteritis Nodosa With Concurrent Perforated Sigmoid Diverticulitis

**DOI:** 10.7759/cureus.21509

**Published:** 2022-01-23

**Authors:** Christopher Leung, Assad Zahid

**Affiliations:** 1 Surgery, Liverpool Hospital, Sydney, AUS; 2 School of Medicine, Western Sydney University, Sydney, AUS; 3 Surgery, Sydney Adventist Hospital, Sydney, AUS

**Keywords:** polyarteritis, sigmoid perforation, large bowel perforation, sigmoid diverticulitis, diverticulitis mimic, intestinal perforation, colonic diverticulum, polyarteritis nodosum

## Abstract

Polyarteritis nodosa (PAN) is a medium-sized vasculitis with neuropathy, cutaneous manifestation, and gastrointestinal tract symptoms. An acute surgical abdomen is a severe but rare development of gastrointestinal involvement of PAN, with large bowel involvement and subsequent perforation being sporadic. Here we present a rare case of PAN who had large bowel involvement with perforation due to newly diagnosed PAN, who also had concurrent and separately perforated sigmoid diverticulitis, confusing the clinical picture. High clinical suspicion and timely management are vital in diagnosing and managing patients with PAN and an acute surgical abdomen.

## Introduction

Polyarteritis nodosa (PAN) is systemic necrotizing arteritis involving mainly medium-sized vessels [1-3]. Polyarteritis often manifests with neuropathy or cutaneous changes [3]. Gastrointestinal manifestation can also occur frequently in patients with polyarteritis nodosa [4]. Unfortunately, thirty-one percent of patients present with an acute surgical abdomen, such as bowel perforation, as a result of gastrointestinal manifestation [4,5]. Those with an acute surgical abdomen have an increased chance of relapse and high mortality [2,6]. Bowel perforation often requires a resection followed by glucocorticosteroids and cyclophosphamide [2,4,7,8].

Large bowel involvement in PAN is rare, with colonic perforation even rarer. Large retrospective studies on PAN have only shown a couple of cases of isolated colonic perforation over many years [2,4]. Most patients appear to have had a surgical resection, and however, follow-up does not appear to be adequately described.

Here we describe a rare case of PAN involving the sigmoid colonic diverticulitis. Given the similar presentation and dual pathology in this patient, a high clinical suspicion and revisiting histopathology is vital in ensuring patients have the correct diagnosis.

## Case presentation

**Figure 1:**
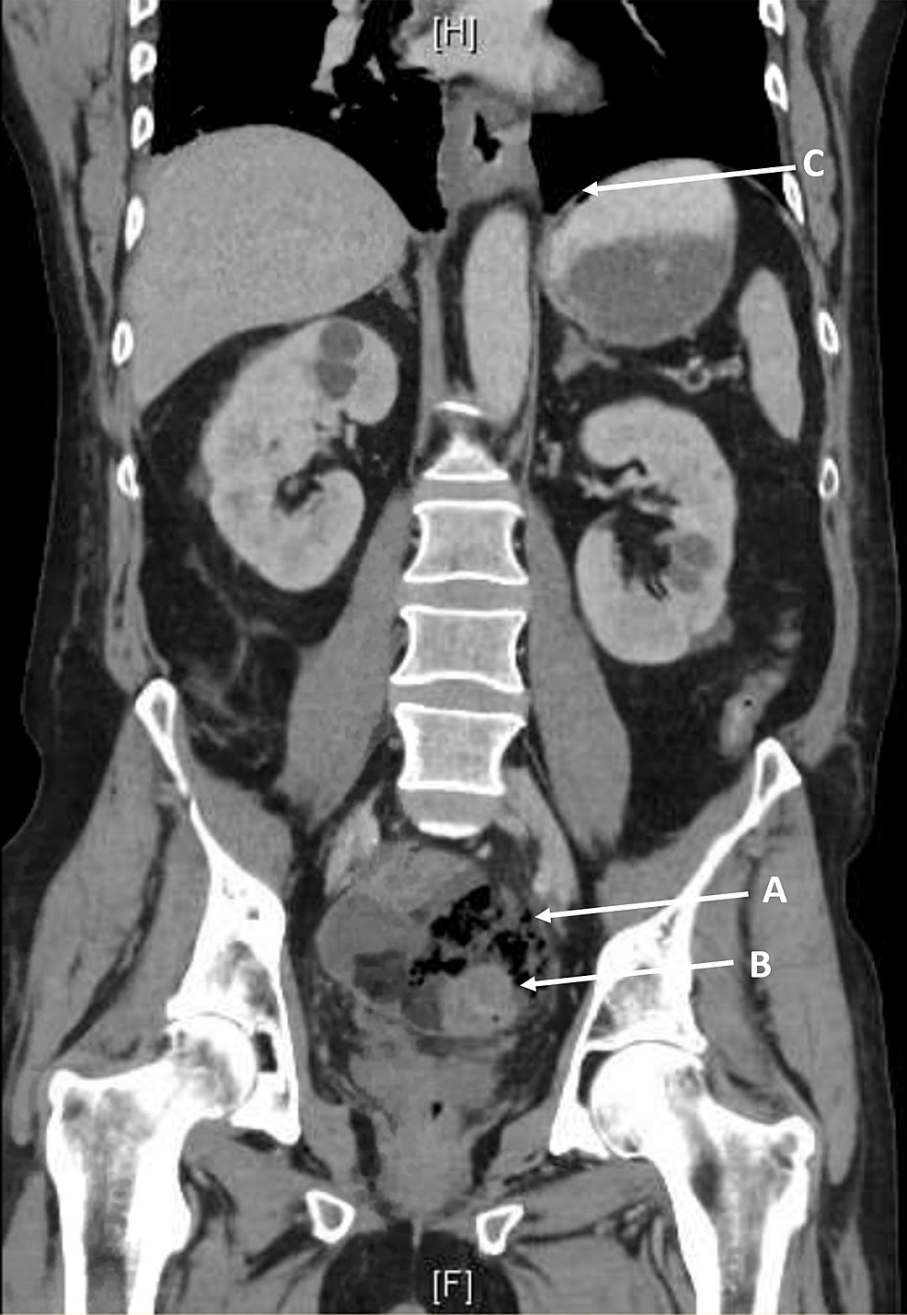
Coronal CT Abdomen and Pelvis Initial CT abdomen and pelvis on presentation to Emergency Department with A = extraluminal gas showing perforation, B = thickened rectosigmoid junction, and C = gas under diaphragm

A 53-year-old male had three days of right-sided abdominal pain before presenting to our hospital’s emergency department. He had nausea without vomiting, subjective fevers, and was obstipated for three days prior to admission. On arrival, the patient had new-onset atrial fibrillation with a tachycardic up to 144 and without any recorded fevers. He had a surgical abdomen with rebound and percussion tenderness to the right abdomen on examination. He had a background of Rheumatoid Arthritis, diagnosed 10 years ago, and Hypertension; and was on 30mg of Prednisone daily, Methotrexate 5mg daily, and Telmisartan 80mg daily. His White Cell Count was 33 x10^9/L, his C-reactive protein was 200mg/L, and his lactate on Venous Blood Gas was 1.5mmol/L; his creatinine and Estimated Glomerular Filtration Rate were normal. A CT abdomen and pelvis showed a perforated sigmoid colon with free fluid and gas next to the sigmoid colon and widespread luminal thickening of the small and large bowel (Figure 1).

Concurringly, this gentleman also complained about joint pain. He had two years of inflammatory joint pain which was managed with Prednisone. However, three months ago, he began to notice bilateral foot numbness and erectile dysfunction. Two weeks prior to his presentation to our Emergency Department, he noticed numbness to his wrist and ankle bilaterally. Before the presentation, he noticed pain and weakness in his hands and feet bilaterally. He did not have any changes to his skin. 

Based on clinical and radiological findings, he had an acute surgical abdomen, and he was taken immediately for an exploratory laparotomy. Intraoperatively, he was found to have perforated sigmoid diverticulitis with four-quadrant purulent peritonitis. Separately, two areas of full-thickness necrosis on the left lateral and right posterolateral aspect of the proximal rectum were found with fecal contamination. An open Hartmann’s procedure was performed with a stapled rectal stump and a distal end colostomy. A thorough washout was performed before his abdomen was closed in layers.

He was taken to our intensive care unit intubated but did not require any inotropes. On day one of his intensive care unit admission, he was extubated and was stepped down to the ward on day two of his admission. He was given stress dose IV hydrocortisone 50mg QID and methotrexate was withheld. Intravenous rhythm controlled therapy was given for two days before being commenced on oral maintenance rate control therapy once the patient returned to sinus rhythm. He progressed well with minimal abdominal pain, and his diet upgraded to a full ward diet on day four of his admission.

While he recovered well from his surgical course, he voiced that his numbness has progressed from his hands to his forearm bilaterally. He was found to have glove stocking sensorimotor peripheral neuropathy. His nerve conduction study showed length-dependent axonal sensorimotor neuropathy with superimposed left ulnar, suggesting mono neuritis multiplex. Rheumatology and Neurology teams were involved, and the patient was immediately commenced on intravenous immune globulin, high dose corticosteroids, and cyclophosphamide.

**Figure 2:**
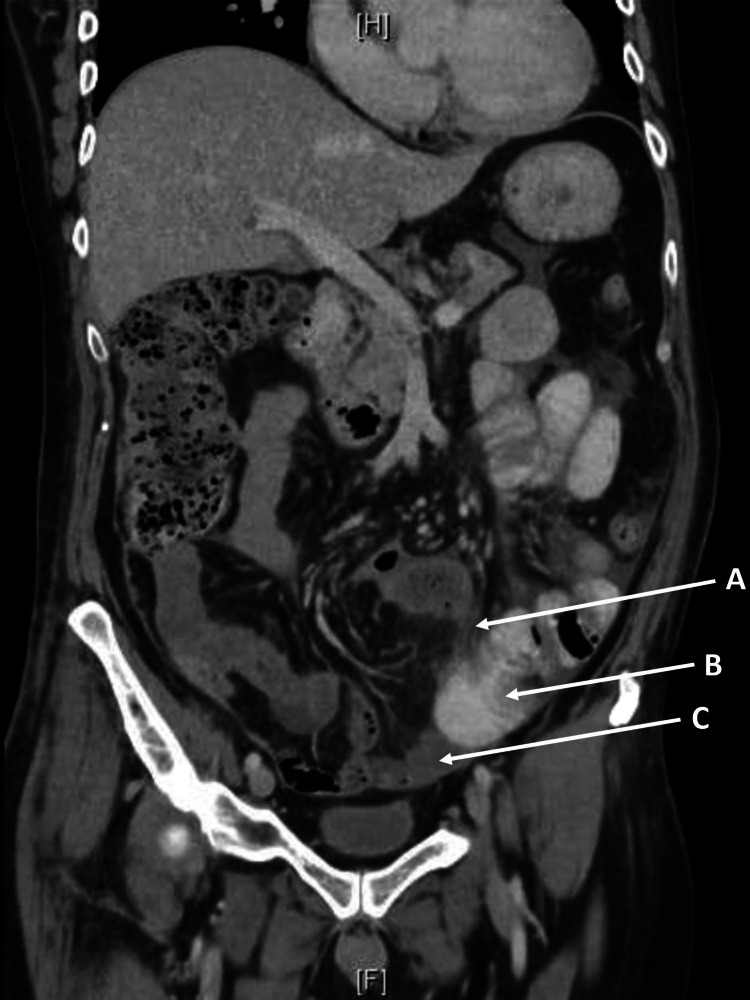
Coronal CT Abdomen and Pelvis CT abdomen and pelvis day 10 post-Hartmann’s procedure with ongoing A = mesenteric vasculitis, B = resolved colitis, and C = free peritoneal fluid.

However, the patient had increasing inflammatory markers on day 10 post-operation, prompting a CT Mesenteric Angiogram (Figure 2). Given the lack of abdominal pain, lack of fevers, and the new diagnosis of mononeuritis multiplex, the mesenteritis was thought to be due to vasculitis. Re-review of the histology, and there was occasional medium-sized acute vasculitis further away from the diverticulitis-associated inflammatory and necrotic changes suggestive of primary vasculitis independent of the diverticulitis. The patient was given the diagnosis of polyarteritis nodosa. The patient has then commenced Prednisolone 75mg and IV cyclophosphamide 750mg fortnightly, with ongoing neuropathy and inflammatory markers improvement. While having ongoing rehabilitation, the patient was discharged day 38 post-operation, on weaning Prednisolone doses and ongoing fortnightly IV cyclophosphamide 750mg, with normalized inflammatory markers and improving neuropathy. Ongoing review at our hospital clinic showed that he has had remission of his neuropathy and is having ongoing follow up with the Colorectal clinic for consideration of reversal of colostomy.

## Discussion

Polyarteritis nodosa (PAN) is a systemic necrotizing vasculitis involving medium-sized arteries. Historically, PAN was associated with prior Hepatitis B Virus (HBV) infection. However, with an increase in the prevention of HBV infection, diagnosis of HBV-associated PAN is becoming increasingly rare with proportionally increased diagnosis of idiopathic PAN [1,3,4,9]. Idiopathic PAN is thought to be autoimmune as the clinical course is arrested with immunosuppression [3,10]. The American Society of Rheumatology has recommended diagnosing PAN based on clinical suspicion in tandem with tissue biopsy or angiography if tissue biopsy cannot be obtained [1]. PAN can involve all organs, including the brain, eyes, pancreas, testicles, ureters, breasts, and ovaries, but half of all cases can affect the gastrointestinal tract. The mean age of diagnosis of PAN was 51.2 years, and most patients frequently present with general symptoms, neurological symptoms, followed by skin involvement [3]. Abdominal pain is the next common symptom accounting for 35.6% of cases [3] and usually occurs within three months of diagnosis [2]. Other gastrointestinal symptoms include nausea, vomiting, diarrhea, haematochezia, melena, and haematemesis [4].

Thirty-four percent of patients have gastrointestinal involvement presenting as peritonitis, bowel perforations, bowel ischemia/infarction, intestinal occlusions, acute appendicitis, cholecystitis, and acute pancreatitis [4]. Unfortunately, patients with gastrointestinal involvement are more likely to relapse 26% of patients die due to an acute surgical abdomen [2,6]. Gastrointestinal involvement (specifically peritonitis, bowel perforation, gastrointestinal ischemia or infarctions, and intestinal occlusion) decreases the life expectancy of patients with polyarteritis nodosa with 6-month and 5-year survival rates of 60% and 46%, respectively [2]. However, survival rates are probably higher in a modern setting due to prompt surgical intervention [2,4].

Treatment of idiopathic PAN includes high-dose corticosteroids and possibly an immunosuppressant depending on the severity of PAN [9]. Sixty percent of PAN without major organ involvement can be induced into remission with glucocorticosteroids alone, while the rest of cases require the addition of azathioprine or methotrexate. Patients with active PAN and major organ involvement require a combination of glucocorticosteroids and cyclophosphamide. Intravenous pulse glucocorticosteroids can be given for up to 3 days at 0.25-1g per day; otherwise, 1mg/kg/day oral prednisone-equivalent (with a maximum of 80mg/day) is given for 2-3 weeks with a gradual taping regimen. Cyclophosphamide is given orally 2mg/kg/day (with a maximum dose of 200mg/day) or intravenous pulses at 15mg/day (with a maximum of 1200mg/pulse) every two weeks for three weeks and then every three weeks after that. Once remission has been achieved, with normalized clinical features and inflammatory markers for 3-6 months, patients can be transitioned from cyclophosphamide to either azathioprine (2mg/kg/day with a maximum of 200mg/day) or methotrexate (20-25mg/week) for a minimum of 18 months [9]. The current survival rate has improved markedly due to corticosteroids and cyclophosphamide with a 1-year and 10-year survival rate of 85% and 80%, respectively [7,9].

Diverticulitis is the infective inflammation of diverticulosis that outpouches within the large bowel consisting of only mucosa and submucosa. The descending and sigmoid colon is the most common site of acute colonic diverticulitis. Classical manifestation of acute colonic diverticulitis includes obstipation and abdominal pain localized to the left lower quadrant. At the same time, free perforation has peritoneal irritation, marked abdominal tenderness that may spread to the rest of the abdomen. The severity of diverticulitis is often graded with Hinchey criteria. Treatment is usually antibiotics for uncomplicated localized infection with up to a small abscess (<3cm), while percutaneous drainage or operative intervention is required for higher staged diverticulitis [11]. Immunosuppression worsens the outcome of diverticulitis by increasing the perforation rate and increasing the severity of perforated diverticulitis.

Large bowel involvement in PAN ranges from colitis, colonic ulcer, colonic ischemia, and colonic perforation [2,5,12]. While 40-60% of patients with PAN have gastrointestinal involvement [2], isolated involvement and subsequent perforation of the colon are rare. Kronzer et al. describe 20 cases of GI perforation with vasculitis from 1998 to 2017 at the Mayo Clinic, but only one patient with PAN had isolated sigmoid perforation [5]. Pragnoux et al. describe 12 cases of PAN with the acute surgical abdomen in France from 1981 to 2002, two had colonic involvement, but none had colonic perforation [2]. Okada et al. describe seven cases with PAN and isolated colonic perforation [8]. In all cases of colonic perforation, the surgical management was not well described. The authors mention an operation in many cases but did not mention whether a colectomy was performed. Most authors did not describe post-operative surgical management, including reversing colostomies, but described patients continuing corticosteroids and/or cyclophosphamide [2,5,8]. Most cases were reported a couple of decades ago, and with the improvement in outcomes with cyclophosphamide, perhaps we may see further reports of long-term follow-up management.

## Conclusions

PAN is a rare medium size vasculitis that frequently has gastrointestinal manifestation. Colonic involvement, however, is uncommon, with colonic perforation even rarer. Patients with PAN and an acute surgical abdomen have a poor prognosis, which is also reflected in colonic perforation. We present a rare case of colonic involvement and perforation with concurrent diverticulitis in the context of PAN, who thankfully, with time management, progressed well.

Both colonic perforations in the context of PAN and perforated diverticulitis without PAN may present with similar presentation initially. Indeed, mesenteric vasculitis and necrosis could worsen diverticulitis, especially when the patient was already on corticosteroids. However, the post-operative management of a patient with PAN is different from that without PAN. Severe PAN with at least one major organ involvement, especially gastrointestinal involvement, should be treated with corticosteroids and cyclophosphamide. Given the similar presentation and dual pathology in this patient, a high clinical suspicion and revisiting histopathology is vital in ensuring patients have the correct diagnosis.

## References

[1] Chung SA, Gorelik M, Langford CA (2021). 2021 American College of Rheumatology/Vasculitis Foundation Guideline for the Management of Polyarteritis Nodosa. Arthritis Rheumatol.

[2] Pagnoux C, Mahr A, Cohen P, Guillevin L (2005). Presentation and outcome of gastrointestinal involvement in systemic necrotizing vasculitides: analysis of 62 patients with polyarteritis nodosa, microscopic polyangiitis, Wegener granulomatosis, Churg-Strauss syndrome, or rheumatoid arthritis-associated vasculitis. Medicine (Baltimore).

[3] Pagnoux C, Seror R, Henegar C (2010). Clinical features and outcomes in 348 patients with polyarteritis nodosa: a systematic retrospective study of patients diagnosed between 1963 and 2005 and entered into the French Vasculitis Study Group Database. Arthritis Rheum.

[4] Levine SM, Hellmann DB, Stone JH (2002). Gastrointestinal involvement in polyarteritis nodosa ( 1986-2000): presentation and outcomes in 24 patients. Am J Med.

[5] Kronzer VL, Larson DP, Crowson CS, Warrington KJ, Ytterberg SR, Makol A, Koster MJ (2019). Occurrence and aetiology of gastrointestinal perforation in patients with vasculitis. Clin Exp Rheumatol.

[6] Eleftheriou D, Dillon MJ, Tullus K (2013). Systemic polyarteritis nodosa in the young: a single-center experience over thirty-two years. Arthritis Rheum.

[7] Ebert EC, Hagspiel KD, Nagar M, Schlesinger N (2008). Gastrointestinal involvement in polyarteritis nodosa. Clin Gastroenterol Hepatol.

[8] Okada M, Konishi F, Sakuma K, Kanazawa K, Koiwai H, Kaizaki Y (1999). Perforation of the sigmoid colon with ischemic change due to polyarteritis nodosa. J Gastroenterol.

[9] Pagnoux C, Mendel A (2019). Treatment of systemic necrotizing vasculitides: recent advances and important clinical considerations. Expert Rev Clin Immunol.

[10] De Virgilio A, Greco A, Conte M, De Vincentiis M (2016). Reply to comment on "Polyarteritis nodosa: a contemporary overview". Autoimmun Rev.

[11] Jacobs DO (2007). Diverticulitis. N Engl J Med.

[12] Gullichsen R, Ovaska J, Ekfors T (1991). Polyarteritis nodosa of the descending colon. case report. Eur J Surg= Acta chirurgica.

